# *SOD2 *polymorphisms: unmasking the effect of polymorphism on splicing

**DOI:** 10.1186/1471-2350-8-7

**Published:** 2007-03-01

**Authors:** Jing Shao, Lishan Chen, Brian Marrs, Lin Lee, Hai Huang, Kenneth G Manton, George M Martin, Junko Oshima

**Affiliations:** 1 Department of Pathology, University of Washington, Seattle, Washington, USA; 2 Center for Demographic Studies, Duke University, Durham, North Carolina, USA

## Abstract

**Background:**

The *SOD2 *gene encodes an antioxidant enzyme, mitochondrial superoxide dismutase. *SOD2 *polymorphisms are of interest because of their potential roles in the modulation of free radical-mediated macromolecular damage during aging.

**Results:**

We identified a new splice variant of *SOD2 *in human lymphoblastoid cell lines (LCLs). The alternatively spliced product was originally detected by exon trapping of a minigene in order to examine the consequences of an intronic polymorphism found upstream of exon 4 (nucleotide 8136, 10T vs 9T). Examination of the transcripts derived from the endogenous loci in five LCLs with or without the intron 3 polymorphism revealed low levels of an in-frame deletion of exon 4 that were different from those detected by the exon trap assay. This suggested that exon trapping of the minigene unmasked the effect of the 10T vs 9T polymorphism on the splicing of the adjacent exon.

We also determined the frequencies of single nucleotide polymorphisms in a sample of US African-Americans and non-African-Americans ages 65 years and older who participated in the 1999 wave of the National Long Term Care Survey (NLTCS). Particularly striking differences between African-Americans and non-African-Americans were found for the frequencies of genotypes at the 10T/9T intron 3 polymorphism.

**Conclusion:**

Exon trapping can unmask *in vitro *splicing differences caused by a 10T/9T intron 3 polymorphism. Given the recent evidence that *SOD2 *is in a region on chromosome 6 linked to susceptibility to hypertension, it will be of interest to investigate possible associations of this polymorphism with cardiovascular disorders.

## Background

The free radical theory of aging is currently the leading hypothesis for a fundamental mechanism of aging. It potentially unifies aging mechanisms across an array of species [1-5]. The theory postulates that reactive oxygen species (ROS) such as superoxide and hydroxyl radicals, mainly generated as toxic by-products of normal cellular metabolism during aerobic respiration in mitochondria, give rise to damage to wide variety of macromolecules, including DNA. Animals possess enzymes that scavenge ROS. Among the most important of such antioxidant defenses are the superoxide dismutases (SOD), which facilitate the dismutation of superoxide free radical (O_2_^-^.) [6]. There are three types of SODs in higher eukaryotes, a copper/zinc containing cytoplasmic enzyme, SOD1; a manganese containing mitochondrial enzyme, SOD2; and a copper/zinc containing SOD3 that is prevalent in extracellular spaces [6,7].

SOD2 is constitutively expressed in most cells [8]. *SOD2 *gene dosage correlates with longevity in fruit flies and in mice [9]. Mice that are heterozygous for an *SOD2 *knockout accumulate excessive damage to mitochondrial DNA as they age [10]. In these mouse lines, there were no compensatory increases of other antioxidant enzymes such as SOD1, catalase or glutathione peroxidase [11]. There is little understanding of the role of SODs in the modulation of longevity or health status in aging humans. Human disorders due to the *SOD2 *mutations have yet to be identified. However, a number of studies describe associations of *SOD2 *polymorphisms (particularly Ala16Val) with age-related disorders. One such study of elderly people with an Ashkenazi ethnic background suggested a possible association between Val at codon 16 of *SOD2 *and longevity [12]. Individuals who were homozygous for the valine allele at position 16 (also described as the -9 position of the mitochondrial targeting sequence) were shown to have an increased risk of non-small cell lung cancer [13,14]. The Val allele may also be a predisposing factor for diabetic polyneuropathy (DPN) in Russians with type 1 diabetes mellitus [15]. Finally, homozygosity for the Val allele was also associated with non-familial idiopathic dilated cardiomyopathy among Japanese [16]. This was thought to be due to a decreased efficiency of SOD2 transport into mitochondria [16].

In contrast to the above reports, the alanine allele has been associated with high risk of breast cancer among U.S. and Finnish Caucasians [17,18] and with an increased risk of developing psoriatic arthritis in the Taiwanese population [19].

As is typical of such association studies, most have not been confirmed in independent populations. Linkage studies are therefore of interest. A particularly cogent example is a report of a genome scan of microsatelite markers among hypertensive African-Americans; linkage was described at 6q24 and 21q21 [20], with Z scores, respectively, of 4.14 and 4.34. Markers associated with hypertension in African-Americans are between 112 cM and 166 cM of 6q. SOD2 is located at 160 cM.

The National Long-Term Care Survey (NLTCS) reports on the functional status of cross-sectional and longitudinal population-based samples from U.S. Medicare recipients ≥ age 65 [21]. During the 1999 wave of that survey, biological samples were obtained. This provided an opportunity to examine *SOD2 *polymorphisms among different ethnic groups.

## Results

### The 1999 NLTCS populations

DNA was obtained from a total of 644 blood samples. Each DNA isolation gave an average of 40 ug of DNA, sufficient for 800 PCR reactions. We also received 2,102 buccal wash samples of which 2,078 provided DNA suitable for banking. There was sufficient DNA from buccal wash samples to permit the performance of up to ~400 PCR reactions.

Screening studies were performed using genomic DNA from peripheral blood. Because a striking difference in the frequencies for the polymorphism at the nucleotide position 8,136 was observed among African-Americans (see below), studies of that polymorphism were extended to the larger sample of genomic DNA from buccal samples.

### SOD2 polymorphisms

We first sequenced exons 1 through 4 and the coding regions of exon 5, including approximately 50 nucleotides into the introns from the exon-intron junctions (Table 1). Exons 1 and 2 were amplified in one amplicon, and exon 1 with the adjacent intron (including a A/G polymorphism at nt 36) was sequenced with the forward primer, and exon 2 with the reverse primer.

**Table 1:** SOD2 primer sequences and PCR conditions used for this study

SOD2 exon	Primers (Top-forward primer, Middle-reverse primer, Bottom-sequencing primer)	Size of amplicon	Tm (C°)	Mg^2++ ^(mM)
1&2	A-GTAGCACCAGCACTAGCAGGA	713	55	2
	B-AAGCGAGTTCTCCTCCCGGAGA			
	P-GTCCACTGTCGCCATTG (for exon 1)			
	Q-GTGGTACGCTGACTGAC (for exon 2)			
3	K-CCAGGTGTCGCATTCTGATGTTG	362	55	2
	D-CAGTAGAGCATCTCTCCCAAATG			
	C-GAAATCTGTTCATTTGTGGGTGG			
4	E-GCTGGTCCCATTATCTAATAGC	277	55	2
	F-CAATCGATTCCTACTGTGCAC			
	L-CAGTGGTTGAAAAAGTAGGAG			
5	G-TTAGACTGAAACTGATGGTTGG	217	55	2
	H-CATCTCAGCATAACGATCGTGG			
	M-GCAAGCCATGTATCTTTCAG			

**Figure 1:**
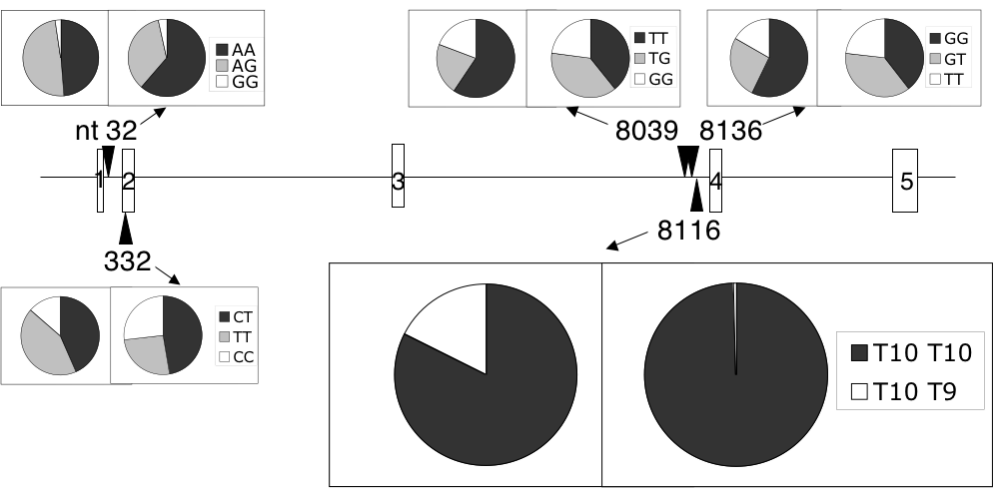
Diagram of the locations of *SOD2 *polymorphisms investigated in this study. The rectangular boxes indicate five exons, with exon numbers. The locations of polymorphisms examined in this study are show in triangles with nucleotide numbers. Circular charts presents the frequencies of genotypes in African-Americans (left) and Non-African Americans (right) also shown in table 2. Nucleotide numbers correspond to the current one in GenBank accession number NT_007422.12 (2390513...2401544), GI: 29803241.

We identified five *SOD2 *polymorphisms, defined as those with minor allele frequencies that were greater than 1% (Table 2, Figure 1). In addition to these polymorphisms, we observed one individual heterozygous for a synonymous change, T to A, at nt 10574 (A188A); one individual heterozygous for a C-to-G change at nt 10577 (Y188X); one individual with a heterozygous A-to-G change at nt 10591 (K194R); one individual heterozygous for a T-to-G change at nt 10595 (N195K), and one individual with a G insertion at 10604 (resulting in the frame shift and premature termination at amino acid 214). We confirmed these five heterozygous changes by sequencing of the reverse strands. One or more of these may prove to be mutations. No other alterations were observed.

**Table 2:** Frequencies of investigated *SOD2 *polymorphisms in the 1996 NLTCS blood samples (N = 613–639).

Nucleotide Position	Location	Genotype	African-American	White
36	Intron 1	A/A	19 (48.7%)	352 (61.9%)
		A/G	19 (48.7)	198 (34.8%)
		G/G	1 (2.6%)	19 (3.3%)
		Total	39 (100%)	569 (100%)
		H-W E	0.137	0.183
		P	0.212	
332	Exon 2	C/C*	5 (13.5%)	156 (27.2%)
		C/T*	16 (43.3%)	270 (47.1%)
		T/T*	16 (43.3%)	147 (25.7%)
		Total	42 (100%)	573 (100%)
		H-W E	0.755	0.184
		P	0.044	
8039	Intron 3	T/T	25 (59.5%)	231 (39.0%)
		T/G	9 (22.4%)	225 (38.0%)
		G/G	8 (19.1%)	136 (23.8%)
		Total	42 (100%)	592 (100%)
		H-W E	0.0016	<0.0001
		P	0.031	
8116	Intron 3	G/G	24 (57.1%)	232 (39.3%)
		G/T	11 (26.2%)	222 (37.6%)
		T/T	7 (16.7%)	137 (23.2%)
		Total	42 (100%)	591 (100%)
		H-W E	0.015	<0.0001
		P	0.093	
8134	Intron 3	T10/T10	36 (83.7%)	592 (99.3%)
		T10/T9	7 (18%)	4 (0.7%)
		T9/T9	0	0
		Total	43 (100%)	592 (100%)
		H-W E	0.544	0.934
		P	< 0.0001	

Nucleotide numbering is according to the sequence of GenBank accession number NT_007422.

H-W E are p values of Hardy-Weinberg equilibrium. P values show the probability of identical distribution between African-American and Non-African American groups.

*C-to-T change will result in alanine (GCT) to valine (GTT) substitution at amino acid 16.

### Is the intron 3 T9/T10 polymorphism associated with splicing variations?

**Figure 2:**
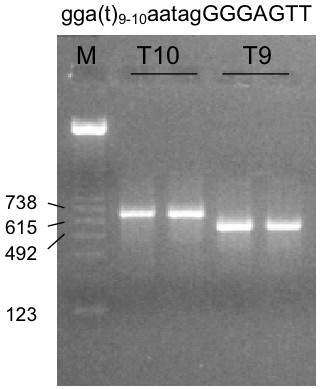
Exon trapping assay of *SOD2 *intron 2 – exon 5 region. Ethidium bromide staining of the RT-PCR products from the exon trapping of the polymorphic genomic fragments (containing either T10 and T9 polymorphism). M is the marker lane with described sizes.

Among the five polymorphisms studied, a single base pair deletion at nucleotide 8,136 was designated as T9 and the wild type was designated as T10. Since the T10/T9 polymorphism is close to the intron-exon junction, we explored the possibility that this polymorphism may affect the splicing of exon 4. An approximately 3 kb fragment spanning intron 2 to exon 5 was inserted into an exon trap system to determine how the fragments were processed. RT-PCR showed that the minigene containing T9 was shorter than expected by approximately 100 bp (Figure 2). Sequencing of the RT-PCR products revealed that the spliced product from the T9 minigene deleted exon 4. Exon 3 was joined directly to exon 5. The RT-PCR product generated from the minigene containing the T10 yielded wild type product, exon 3 being joined to exon 4 followed by exon 5.

**Figure 3:**
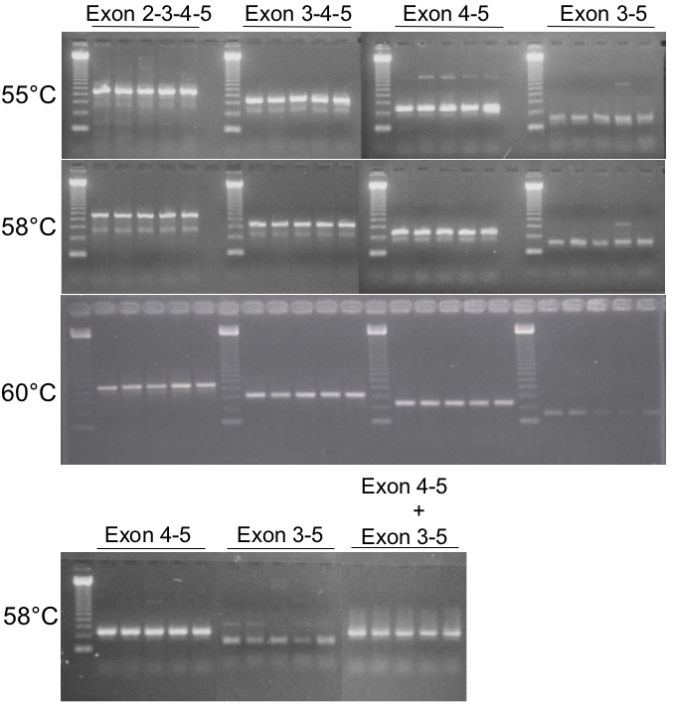
RT-PCR detection of SOD2 mRNAs. Primer sets were designed RT-PCR product spanning exon 2 through 5 (2–3–4–5), exon 3 through 5 (3–4–5), exon 4 to 5 (4–5) and exon 3 to 5 deleting 4 (3–5). Each set are tested in two T10/T10 homozygotes (first 2 lanes) and three T10/T9 heterozygotes (next 3 lanes) at three different annealing temperatures, 55°C, 58°C, and 60°C. Bottom panel shows the RT-PCR products with the primer sets that detect exon 4–5 only, 3–5 only and the combination of 4–5 plus 3–5.

Exon trapping, however, is a highly artificial system. The splicing of the minigene occurs out of context of the complete genome. In order to examine whether deletion of exon 4 can be spliced out in intact cells, lymphoblastoid cell lines were established from four T10 homozygotes and four T10/T9 heterozygotes. *SOD2 *mRNAs from these cell lines were amplified by RT-PCR. We utilized one primer set that amplifies only those mRNA deleting exon 4 and another set which amplifies both wild type and the exon 4-deleted form (Figure 3). Ethidium bromide staining of RT-PCR products showed that LCLs from both homozygous 10T and heterozygous T9/T10 subjects were expressing wild type SOD2 mRNA and the aberrant form deleting exon 4 to similar extents. These RT-PCR products were sequenced to confirm that they are all SOD2 mRNA, with or without exon 4. When both forms were simultaneously amplified, only wild type mRNA was detected, indicating that the predominantly spliced forms were wild type in cells from both genotypes. These results suggest that the exon-trapping assay excluded *cis*-acting factors required for the inclusion of exon 4 when one T is missing at the IVS4-6 position.

### Dramatically altered 8136 allele frequencies within the African-American population

The 644 blood samples were derived from 596 Caucasians, 43 African-Americans, 2 Asian/Pacific islanders, and 3 "others". Statistically significant differences between African-Americans and Caucasians were observed for three out of five polymorphisms. At the Ala16Val polymorphism in exon 2 (which has been reported to alter the risk of several disorders), the Val (GTT) was more frequent than Ala (GCT) in the African-American population (P = 0.044). The C8039G polymorphism in intron 3 also showed a statistically significant difference between African-Americans and Caucasians (P = 0.031). A statistically significant difference was not observed for the case of the adjacent G8116T polymorphism (P = 0.093).

Among the five polymorphisms studied, the most striking finding was a difference in the frequencies of the polymorphism at nucleotide 8,136 (Table 2). A single base pair deletion was designated as T9 and the wild type designated as T10. Among 40 African-Americans, 18% carried one T9 allele, significantly higher than the average for the total population (1.7%) and strikingly higher when compared to the population of non-African-Americans (0.7%). We then examined this T9/T10 polymorphism in buccal samples that were obtained from African-Americans. Out of 67 African-Americans who provided buccal wash samples, 54 (81%) were T10/T10 and 13 (10%) were T10/T9. No individual has been found to be homozygous for the T9 allele. The allele frequencies from blood and buccal samples were comparable. We concluded that the T9 allele is far more frequent in elderly African-Americans than in non-African-Americans (*p *< 0.0001).

## Discussion

The T9/T10 *SOD2 *polymorphism was newly identified in our laboratory in the course of screening of the 1999 NLTCS population for *SOD2 *polymorphisms. Our analysis indicated that the T9 allele is highly prevalent in elderly African-Americans as compared to elderly Caucasian-Americans (*p *< 0.0001). The location of T9/T10 at an intron/exon boundary region suggested a role in pre-mRNA processing. An exon trap analysis was quite consistent with this interpretation. There are a number of examples in the human genetics literature in which intron polymorphisms appear to have altered the ratio of wild type and alternative splicing patterns [22,23]. In some cases, altered splicing was shown be responsible for the pathogenetic mechanism leading to disease. Interesting examples are the sequence variations at the 3' end of intron 8 of cystic fibrosis transmembrane conductance regulator (CFTR). These are thought to affect splicing of exon 9, resulting in the reduction of functional CFTR [24]. For the case of our SOD2 T9/T10 polymorphism, however, it was not possible to confirm the splicing bias using RNA from intact cells (lymphoblastoid cell lines). Similarly, we were unable to determine what fractions of the transcript lacking exon 4 were derived from T10 and T9 alleles in the heterozygote.

Previous work from our laboratory showed that intronic polymorphisms generated cryptic splicing products by exon trapping or by assays utilizing a mini gene and RT-PCR, but there was little evidence that this occurred *in vivo *[25]. The most likely interpretation of this discordance in the results of test tube-based versus cell-based methods is that the former exclude important cis-acting domains residing in more distal domains of DNA. In addition, trans-acting factors may differ among different cell types and species. These elements are likely to be capable of modulating splicing.

Although we have so far failed to provide molecular evidence for a functional distinction between the polymorphic allele of interest, given the striking difference in prevalence among African-Americans as compared to Caucasian-Americans, it will be important to carry out appropriate epidemiological studies of the impact of the two alleles (and, eventually, contrasting haplotypes), upon health and survival in the two ethnic groups. The NLTCS should provide valuable material for such an investigation, as there is linkage to Medicare data and data on functional status and survival. A definitive study, however, must await the availability of biological material from future cohorts, as the 1999 wave of the study could not provide biological specimens from many institutionalized subjects.

Perhaps the most immediate question would be the potential impact of this allelic variation of *SOD2 *upon susceptibility to hypertension among African-Americans. First, hypertension is pervasive in this population. Secondly, it has deleterious effects upon multiple organs. Third, while susceptibility to hypertension is clearly modulated by multiple genetic loci, a region of chromosome 6 that encompasses the *SOD2 *locus is among those that have been implicated [26]. Fourth, African-Americans provide an excellent opportunity to carry out admixture mapping (reviewed by [27,28]. It has been estimated that ~75% of the alleles of African-Americans are of African ancestry whilst ~25% are of European ancestry [29]. Gene scans on the parental and admixed populations were successfully employed to identify hypertension loci by Zhu et al. (2005). The most robust finding was a region on the long arm of chromosome 6 that included the *SOD2 *locus. While the region contains many genes, potentially including more than one susceptibility genes, our observations suggest that one important candidate gene is *SOD2*.

## Conclusion

We demonstrated that the 10T/9T polymorphism in the *SOD2 *intron 3 clearly results in alternative splicing of exon 4 by an *in vitro *exon trap assay using minigenes. This difference was masked when endogenous genes were expressed within cells. Over-representation of 9T alleles in African-Americans nevertheless raises the possibility that this intron 3 polymorphism or polymorphisms in linkage disequilibrium may be involved in the altered susceptibilities to a range of disorders that are more common in African-American populations.

## Methods

### Sample collection and DNA isolation

Both peripheral blood and buccal wash samples were obtained from the 1999 NLTCS participants. Informed consent was obtained. Peripheral blood mononuclear cells were isolated by Ficoll-Hypaque gradients (Amersham, CA, USA) gradient. Buccal wash samples were centrifuged at 3,000 × g prior to DNA isolation. Genomic DNA from blood and buccal wash samples were isolated with QIAamp blood isolation kits or QIAamp tissue isolation kits, respectively (Qiagen Inc. Valencia, CA), following protocols specified by the manufacturer. For high throughput DNA isolation, a Qiagen BioRobot 3000 system was employed for DNA manipulation. Isolated genomic DNA samples were kept in 96 well microtiter plates in duplicates, and stored at -80°C.

### Single nucleotide polymorphism (SNP) detection

The SNPs in *SOD2 *were determined through direct sequencing of *SOD2 *exons and adjacent portions of introns. Briefly, genomic DNA was first amplified by 4 different primer pairs (Table 1), designed using Primer Express software (Applied Biosystems Co., Foster City, CA), to obtain DNA fragments containing exons 1 and 2, exon 3, exon4, and exon 5, respectively. The primer pairs were designed to specifically amplify the designated exon(s) with maximum adjacent upstream and downstream intron sequences. All PCR products were first examined on agarose DNA electrophoresis to verify size and were treated with shrimp alkaline phosphatase and exonuclease I to eliminate excess nucleotides. The subsequent sequencing of the PCR products was performed by a Big Dye-terminator cycle sequencing kit (ABI) on an ABI PRISM 310 automated DNA sequencer with factory-supplied parameters. The sequencing primers for the 4 PCR products are also listed in Table 1. Newly found polymorphisms were confirmed by sequencing in the reverse orientation in all of the samples.

### Statistical analysis

Distributions of allele frequencies within the groups were examined with the chi-square test (one degree of freedom) to test for Hardy-Weinberg equilibrium. Comparisons of the distributions between two groups were tested by the modified Fisher exact test (two degrees of freedom) [30,31]. The latter was also examined with the standard Pearson chi-square test [32].

### Exon trapping

Exon trapping was performed to reveal the potential of SNPs to promote differential splicing of *SOD2 *exons. Genomic fragments of different alleles of *SOD2 *were first amplified with a set of primers (forward: gct ggt ccc att atc taa tag c; reverse: cag tag agc atc tct ccc aaa tg) encompassing *SOD2 *intron 2 to exon 5 by long-range PCR (Roche Diagnostics Co. Indianapolis, IL). The DNA fragments carrying the SNPs in question were cloned by TA cloning techniques (Invitrogen, Hercules, CA), and the SNPs were verified by sequencing. The selected genomic DNA fragments with the intended SNPs were subcloned into a *pSPL3 *splicing vector for exon trapping analysis (Invitrogen Co. Carlsbad, CA). The nucleotide sequences at the polymorphic site were confirmed by sequencing, and the constructs were verified via the digestion patterns of combinations of restriction enzymes. The exon trapping procedures were performed according to the manufacturer's specifications. The genomic DNA in *pSPL3 *was first isolated with a plasmid purification kit (Qiagen), and was used to transfect COS-7 cells for transient expression of the reporter system. Exon trapping reporter transcripts were examined by extracting total RNA from COS-7 cells, followed by the reverse transcriptase polymerase chain reaction (RT-PCR) with primers specified in the kit for amplification of the reporter gene. The amplified reporter gene fragments were visualized on 2% agarose gels. Variable lengths of the amplified fragments revealed differential splicing as affected by the inserted alleles in the reporter system.

### Establishment of lymphoblastoid cell lines and RT-PCR

Cryopreserved lymphocytes were thawed and immortalized with Epstein-Barr virus using a published protocol [33]. Total RNA was isolated using Trizol (Gibco BRL) and was reverse-transcribed with random hexamers with GeneAmp RNA PCR kit (Perkin Elmer Cetus Inc., Wellesley, MA) following the manufacturer's instructions. PCR reactions were performed as described previously [30,31]. The products were separated on a 2% agarose-1xTAE gel.

## Competing interests

The author(s) declare that they have no competing interests.

## Authors' contributions

JS carried out the splicing analysis. LC carried out sequencing analysis and sequence alignment. BM carried out the sequencing analysis. LL participated in the sequencing analysis. HH participated in the statistical analysis. KGM provided sample information. GMM conceived the study. JO coordinated the study and drafted the manuscript. All authors read and approved the final manuscript.

## Pre-publication history

The pre-publication history for this paper can be accessed here:



## References

[B1] Harman D (1956). Aging: A theory based on free radical and radiation chemistry. J Gerontol.

[B2] Harman D (1992). Free radical theory of aging. Mutat Res.

[B3] Finkel T, Holbrook NJ (2000). Oxidants, oxidative stress and the biology of ageing. Nature.

[B4] Hekimi S, Guarente L (2003). Genetics and the specificity of the aging process. Science.

[B5] Schriner SE, Linford NJ, Martin GM, Treuting P, Ogburn CE, Emond M, Coskun PE, Ladiges W, Wolf N, Van Remmen H, Wallace DC, Rabinovitch PS (2005). Extension of murine life span by overexpression of catalase targeted to mitochondria. Science.

[B6] Fridovich I (1995). Superoxide radical and superoxide dismutases. Annu Rev Biochem.

[B7] Landis GN, Tower J (2005). Superoxide dismutase evolution and life span regulation. Mech Ageing Dev.

[B8] Lai CC, Huang WH, Askari A, Wang Y, Sarvazyan N, Klevay LM, Chiu TH (1994). Differential regulation of superoxide dismutase in copper-deficient rat organs. Free Radic Biol Med.

[B9] Tower J (2000). Transgenic methods for increasing Drosophila life span. Mech Ageing Dev.

[B10] Williams MD, Van Remmen H, Conrad CC, Huang TT, Epstein CJ, Richardson A (1998). Increased oxidative damage is correlated to altered mitochondrial function in heterozygous manganese superoxide dismutase knockout mice. J Biol Chem.

[B11] Van Remmen H, Salvador C, Yang H, Huang TT, Epstein CJ, Richardson A (1999). Characterization of the antioxidant status of the heterozygous manganese superoxide dismutase knockout mouse. Arch Biochem Biophys.

[B12] Stessman J, Maaravi Y, Hammerman-Rozenberg R, Cohen A, Nemanov L, Gritsenko I, Gruberman N, Ebstein RP (2005). Candidate genes associated with ageing and life expectancy in the Jerusalem longitudinal study. Mech Ageing Dev.

[B13] Wang LI, Miller DP, Sai Y, Liu G, Su L, Wain JC, Lynch TJ, Christiani DC (2001). Manganese superoxide dismutase alanine-to-valine polymorphism at codon 16 and lung cancer risk. J Natl Cancer Inst.

[B14] Liu G, Zhou W, Park S, Wang LI, Miller DP, Wain JC, Lynch TJ, Su L, Christiani DC (2004). The SOD2 Val/Val genotype enhances the risk of nonsmall cell lung carcinoma by p53 and XRCC1 polymorphisms. Cancer.

[B15] Zotova EV, Chistiakov DA, Savost'ianov KV, Bursa TR, Galeev IV, Strokov IA, Nosikov VV (2003). [Association of the SOD2 Ala(-9)Val and SOD3 Arg213Gly polymorphisms with diabetic polyneuropathy in patients with diabetes mellitus type 1]. Mol Biol (Mosk).

[B16] Hiroi S, Harada H, Nishi H, Satoh M, Nagai R, Kimura A (1999). Polymorphisms in the SOD2 and HLA-DRB1 genes are associated with nonfamilial idiopathic dilated cardiomyopathy in Japanese. Biochem Biophys Res Commun.

[B17] Mitrunen K, Sillanpaa P, Kataja V, Eskelinen M, Kosma VM, Benhamou S, Uusitupa M, Hirvonen A (2001). Association between manganese superoxide dismutase (MnSOD) gene polymorphism and breast cancer risk. Carcinogenesis.

[B18] Ambrosone CB, Freudenheim JL, Thompson PA, Bowman E, Vena JE, Marshall JR, Graham S, Laughlin R, Nemoto T, Shields PG (1999). Manganese superoxide dismutase (MnSOD) genetic polymorphisms, dietary antioxidants, and risk of breast cancer. Cancer Res.

[B19] Yen JH, Tsai WC, Lin CH, Ou TT, Hu CJ, Liu HW (2003). Manganese superoxide dismutase gene polymorphisms in psoriatic arthritis. Dis Markers.

[B20] Zhu X, Luke A, Cooper RS, Quertermous T, Hanis C, Mosley T, Gu CC, Tang H, Rao DC, Risch N, Weder A (2005). Admixture mapping for hypertension loci with genome-scan markers. Nat Genet.

[B21] Manton KG, Corder L, Stallard E (1997). Chronic disability trends in elderly United States populations: 1982-1994. Proc Natl Acad Sci U S A.

[B22] Pagani F, Baralle FE (2004). Genomic variants in exons and introns: identifying the splicing spoilers. Nat Rev Genet.

[B23] Chu CS, Trapnell BC, Curristin S, Cutting GR, Crystal RG (1993). Genetic basis of variable exon 9 skipping in cystic fibrosis transmembrane conductance regulator mRNA. Nat Genet.

[B24] Hu Q, Cool BH, Wang B, Hearn MG, Martin GM (2002). A candidate molecular mechanism for the association of an intronic polymorphism of FE65 with resistance to very late onset dementia of the Alzheimer type. Hum Mol Genet.

[B25] Krushkal J, Ferrell R, Mockrin SC, Turner ST, Sing CF, Boerwinkle E (1999). Genome-wide linkage analyses of systolic blood pressure using highly discordant siblings. Circulation.

[B26] McKeigue PM (2005). Prospects for admixture mapping of complex traits. Am J Hum Genet.

[B27] Darvasi A, Shifman S (2005). The beauty of admixture. Nat Genet.

[B28] Destro-Bisol G, Maviglia R, Caglia A, Boschi I, Spedini G, Pascali V, Clark A, Tishkoff S (1999). Estimating European admixture in African Americans by using microsatellites and a microsatellite haplotype (CD4/Alu). Hum Genet.

[B29] Castro E, Edland SD, Lee L, Ogburn CE, Deeb SS, Brown G, Panduro A, Riestra R, Tilvis R, Louhija J, Penttinen R, Erkkola R, Wang L, Martin GM, Oshima J (2000). Polymorphisms at the Werner locus: II. 1074Leu/Phe, 1367Cys/Arg, longevity, and atherosclerosis. Am J Med Genet.

[B30] Castro E, Ogburn CE, Hunt KE, Tilvis R, Louhija J, Penttinen R, Erkkola R, Panduro A, Riestra R, Piussan C, Deeb SS, Wang L, Edland SD, Martin GM, Oshima J (1999). Polymorphisms at the Werner locus: I. Newly identified polymorphisms, ethnic variability of 1367Cys/Arg, and its stability in a population of Finnish centenarians. Am J Med Genet.

[B31] Conover WJ (1980). Practical Nonparametric Statistics.

[B32] Beck JC, Beiswanger CM, John EM, Satariano E, West D (2001). Successful transformation of cryopreserved lymphocytes: a resource for epidemiological studies. Cancer Epidemiol Biomarkers Prev.

